# Variance of matrix metalloproteinase (MMP) and tissue inhibitor of metalloproteinase (TIMP) concentrations in activated, concentrated platelets from healthy male donors

**DOI:** 10.1186/1749-799X-9-29

**Published:** 2014-04-26

**Authors:** Justin M Hire, J Lee Evanson, Peter C Johnson, Steven D Zumbrun, M Kelly Guyton, James C McPherson, John A Bojescul

**Affiliations:** 1 Department of Orthopaedics and Rehabilitation, Dwight D. Eisenhower Army Medical Center, 300 Hospital Road, Fort, Gordon, GA 30905, USA; 2 Department of Clinical Investigation, Dwight D. Eisenhower Army Medical Center, 38th Street, 7th Avenue, BLDG 38705, Fort, Gordon, GA 30905, USA

**Keywords:** Platelet-rich plasma (PRP), MMP, TIMP, ADAMTS13, Wound healing, Tissue remodeling

## Abstract

**Background:**

The use of autologous blood concentrates, such as activated, concentrated platelets, in orthopaedic clinical applications has had mixed results. Research on this topic has focused on growth factors and cytokines, with little directed towards matrix metalloproteinases (MMPs) which are involved in post-wound tissue remodeling.

**Methods:**

In this study, the authors measured the levels of MMP-2, MMP-9 and a disintegrin and metalloproteinase with a thrombospondin type 1 motif, member 13 (ADAMTS13), in activated platelets derived from blood of healthy, male volunteers (*n* = 92), 19 to 60 years old. The levels of the natural inhibitors of these proteases, tissue inhibitor of metalloproteinase 1 (TIMP-1), TIMP-2 and TIMP-4 were also assessed.

**Results:**

Notably, there was no significant change in concentration with age in four of six targets tested. However, TIMP-2 and TIMP-4 demonstrated a statistically significant increase in concentration for subjects older than 30 years of age compared to those 30 years and younger (*P* = 0.04 and *P* = 0.04, respectively).

**Conclusion:**

TIMP-2 and TIMP-4 are global inhibitors of MMPs, including MMP-2 (Gelatinase A). MMP-2 targets native collagens, gelatin and elastin to remodel the extracellular matrix during wound healing. A decreased availability of pharmacologically active MMP-2 may diminish the effectiveness of the use of activated, concentrated platelets from older patients, and may also contribute to longer healing times in this population.

## Background

The use of activated, concentrated platelets, also known as platelet-rich plasma (PRP), in orthopaedic therapies is well studied. Current indications include treatment of medial and lateral epicondylitis, tendinopathies, muscle injuries, joint osteoarthritis, as well as for use intraoperatively with total joint arthroplasty to decrease blood loss and narcotic requirements [1,2]. This therapy is a simple tool that presumably harnesses the body's natural healing pathways to promote faster repair of injured tissue. However, the efficacy of these treatments has been debated [3-6]. Differences in the amounts and contents of components in PRP depend on the preparation method, and are also a function of human variability [7,8]. Both preparation method and human variability affect PRP efficacy, and this may perhaps explain the mixed results observed in clinical trials [5]. Thus, more consistent positive results may perhaps be achievable if PRP use or dosage is optimized based on content, which may be a function of factors like age or gender.

The concentration of growth factors and cytokines in PRP are well described, some having correlations with age [7,8]. However, the understanding of the dynamic interplay between cell mediators remains incomplete since matrix metalloproteases (MMPs) and tissue inhibitors of metalloproteinases (TIMPs) are also found in platelets and affect the activity of growth factors [9]. MMPs are known modulators of the extracellular matrix through the cleavage of growth factor receptors, cause the release of growth factors, independently affect the extracellular matrix, and regulate all phases of wound healing [9,10]. TIMPs affect tissue remodeling by inhibiting MMPs in a 1:1 inhibitor-to-enzyme ratio but have also been shown to have a direct biological effect on multiple cell lines independent of inhibitor activity [10]. Little is known about the concentrations of MMPs and TIMPs in PRP or how these might vary by age. A disintegrin and metalloproteinase with a thrombospondin type 1 motif, member 13 (ADAMTS13), although not an MMP or TIMP, was included in this study due to recent research that implicates this molecule in hemostasis and wound healing [11]. This study was designed to determine the concentrations of MMPs and TIMPs in standardized, activated platelet concentrates and to correlate these values with age, whole blood thrombocyte counts and PRP thrombocyte counts.

## Methods

### Study design and sample collection

This study was approved by the Institutional Review Board (IRB) at Dwight D. Eisenhower Army Medical Center, Fort Gordon, GA. Blood samples were collected from healthy individuals donating blood during routine blood drives in collaboration with the Kendrick Memorial Blood Donor Center (Fort Gordon, GA). All identifiable subject information was omitted by study personnel. Blood donors were provided with an information sheet before donating blood, which explained the purpose of the study and allowed them to decline participation without affecting their ability to donate blood. Blood samples from a total of 92 males with a mean age of 34.4 ± 9.76 years old (average ± SD, range 19–60) were collected. Only age, gender, time of draw and time of PRP activation were collected.

### Isolation and activation of PRP

Citrated blood collection tubes (BD Biosciences, Franklin Lakes, NJ, USA) were used to collect 8.5 mL of unused blood from the diversion pouch of the blood donor bag only after all tubes necessary for safety testing of donated blood were collected. If 8.5 mL of blood was not available, the tube was discarded and that sample was not included in the study. The collected blood was then drawn from the collection tube into an Arthrex autologous conditioned plasma (ACP) double syringe system (Arthrex Systems, Naples, FL, USA) within 3 h of collection and spun at 1,500 RPM for 5 min in a centrifuge (Hettich Rotofix 32, Andreas Hettich GmbH & Co., Tuttlingen, Germany) as described by the manufacturer. PRP was isolated and transferred to a 15-mL conical tube. Aliquots of whole blood and PRP were measured for red blood cell (RBC), white blood cell (WBC) and platelet concentrations with a Coulter Ac T diff analyzer (Beckman Coulter, Pasadena, CA, USA). The volume of PRP collected was recorded and the PRP activated with 100 U bovine thrombin (Sigma-Aldrich, St. Louis, MO, USA) in 10% calcium chloride (CaCl_2_) (Sigma-Aldrich,) per mL PRP. A solid clot formed immediately. Then an amount of sterile saline (Sigma-Aldrich, St. Louis, MO, USA) equivalent to the volume of PRP collected for each sample was added to the clot, and the clot was gently disrupted to mix the components. After 90 min, the remaining supernatant containing the released MMPs and TIMPS were collected for future analyses. Samples were immediately snap-frozen in liquid nitrogen and stored at −80°C. Aliquots were thawed once to reduce MMP and TIMP degradation.

### MMP and TIMP quantification

A combination of six MMPs and TIMPs were individually analyzed by enzyme-linked immunosorbant assay (ELISA; R&D Systems, Minneapolis, MN, USA). Matrix metalloproteinase-2 (MMP-2), matrix metalloproteinase-9 (MMP-9), tissue inhibitor of metalloproteinase-1 (TIMP-1), tissue inhibitor of metalloproteinase-2 (TIMP-2), tissue inhibitor of metalloproteinase-4 (TIMP 4), and a disintegrin and metalloproteinase with a thrombospondin type 1 motif, member 13 (ADAMTS13) were measured *via* manufacturer's recommendations. Standards and samples were run in duplicate. Any value that was below the limit of detection, based on a standard curve, was assigned the lowest detectable value. This situation occurred in three instances and is described in the ‘Results’ section below.

### MMP activity assay

The MMP activity level in each sample was determined by fluorometric assay (Anaspec, Fremont, CA, USA). A generic MMP substrate was utilized in the assay, in order to measure total activity levels for MMPs generally, that included MMP-1, MMP-2, MMP-3, MMP-7, MMP-8, MMP-9, MMP-12, and MMP-13. Not all of these MMPs are released from platelets [12]. However, this activity assay included the key MMPs being studied and would give a more global perspective of overall MMP activity present in the samples. Relative activity (ra) was determined by measuring the difference between each sample compared to a blank. Eight samples processed in the previous MMP/TIMP ELISA experiments were omitted because of lack of sufficient sample volume. The omitted samples included three from the population of ≤30-year-olds, and five from the population of >30-year-olds.

### Statistical analyses

Statistical analysis was performed in SPSS version 19. Quantitative measurements were described with summary statistics (*n*, mean, standard deviation, median, minimum, maximum, and other quantities). Intra-assay variability was calculated to demonstrate the consistency of results between duplicate samples with 1 being ideal. Two-tailed correlation coefficients were calculated to demonstrate relationships between age and MMP/TIMP concentrations present in PRP (Pearson's correlation coefficient, *r*_p_). The study population was divided into two study groups operationally defined based on a median split (31.5 years). Rather than relying on the actual median, the median split was rounded down to 30 for ease of interpretation. A series of multivariate tests were performed with WBC as a covariate comparing subjects older than 30 years of age and younger to those older than 30 years. The null hypothesis for each test was rejected at *P* ≤ 0.05.

## Results

The platelet count from whole blood was 151 ± 35.3 × 10^3^/μL (mean ± SD). The platelet count from PRP was 248 ± 65.3 × 10^3^/μl. Concentrations of MMPs and TIMPs were found to vary widely (Table 1). Intra-assay variability between duplicates was low with MMP-2 = 0.951, MMP-9 = 0.870, ADAMTS13 = 0.968, TIMP-1 = 0.690, TIMP-2 = 0.936, and TIMP-4 = 0.974. ADAMTS13 had one sample below the detectable limit of 31.729 pg/mL (age = 44 years old) and TIMP-4 had two samples below the detectable limit of 262.78 pg/mL (ages = 21 and 51 years old). Neither of the later was from the same sample as ADAMTS13.

**Table 1:** Descriptive statistical parameters

	**Age (years)**	**MMP-2**	**MMP-9**	**TIMP-1**	**TIMP-2**	**TIMP-4**	**ADAMTS13**
		**(ng/mL)**	**(ng/mL)**	**(ng/mL)**	**(ng/mL)**	**(ng/mL)**	**(pg/mL)**
*N*	92	92	92	92	92	91	92
Mean	34.40	82.46	21.41	46.66	44.71	488.95	333.51
95% CI	[32.28 to 36.42]	[77.73–87.19]	[17.57–25.25]	[44.07–49.25]	[42.44–46.99]	[457.88–520.02]	[312.55–354.47]
Median	31.50	78.50	16.81	45.79	42.61	487.28	33.70
SD	9.76	22.84	18.53	12.52	11.00	149.18	101.22
Minimum	20	15.30	7.69	14.46	9.02	262.78	31.73
Maximum	57	170.65	155.27	81.78	81.43	899.00	757.67
Percentile							
10	23.00	60.32	10.09	32.51	34.79	300.17	226.43
25	26.00	68.80	11.96	38.20	38.03	358.54	288.36
75	42.75	92.46	23.80	54.37	49.00	582.86	376.76
90	49.70	110.41	34.63	62.04	58.53	679.29	432.85

The majority of targets demonstrated statistically significant correlations with one another (Table 2). WBC was not found to significantly correlate with any of the other measures. TIMP-2 had an increased concentration with age with linear regression analysis (*r*_p_ 0.226, *P* = 0.025). TIMP-2 and TIMP-4 demonstrated statistically significant increases for subjects over 30 years of age compared with subjects 30 years of age and younger (46.96 ± 10.85 ng/mL vs 41.80 ± 10.63 ng/mL, *P* = 0.04, and 517.39 ± 149.64 ng/mL vs 451.04 ± 141.69 ng/mL, *P* = 0.04, respectively) (Table 3). MMP-2, MMP-9, ADAMTS13, and TIMP-1 displayed no significant differences in quantity with age (*P* > 0.05). Overall MMP activity decreased with age (≤30-year-olds' average activity = 118 ra, 95% CI = 8.76 vs. >30-year-olds' average activity = 110 ra, 95% CI = 6.96), but not enough to achieve statistical significance by *T* test (*P* = 0.15).

**Table 2:** Pearson's correlation coefficients for age, thrombocyte counts, and matrix metalloproteinases (MMPs)/tissue inhibitor of metalloproteinases (TIMPs)

	**Age**	**MMP-2**	**MMP-9**	**TIMP-1**	**TIMP-2**	**TIMP-4**	**ADAMTS13**
MMP-2	n. s.						
MMP-9	n. s.	0.282*					
TIMP-1	n. s.	0.414*	0.326*				
TIMP-2	0.226*	0.854*	0.261*	0.332*			
TIMP-4	n. s.	0.550*	0.272*	0.256*	0.543*		
ADAMTS13	n. s.	0.526*	n. s.	0.425*	0.457*	0.309*	
PRP WBC	n. s.	n. s.	n. s.	n. s.	n. s.	n. s.	n.s.

*Significant correlation *P* < 0.05; n.s., not statistically significant.

**Table 3:** Analysis of MMP, TIMP, and ADAMTS13 concentrations in relation to age

**Target age (years)**	**Mean (SD)**	***P *** **value**	**Power**	**Effect size**
MMP-2		0.08	0.42	0.04
≤ 30	77.56 (23.82)			
> 30	86.23 (21.54)			
MMP-9		0.11	0.28	0.02
≤ 30	18.24 (11.47)			
> 30	23.85 (22.33)			
TIMP-1		0.15	0.36	0.03
≤ 30	44.25 (12.04)			
> 30	48.52 (12.67)			
TIMP-2		0.04*	0.57	0.05
≤ 30	41.80 (10.63)			
> 30	46.95 (10.85)			
TIMP-4		0.04*	0.56	0.05
≤ 30	451.04 (141.69)			
> 30	517.39 (149.64)			
ADAMTS13		0.50	0.07	0.01
≤ 30	325.40 (118.51)			
> 30	339.75 (86.32)			

Analysis of concentrations of metalloproteinases (MMPs) and tissue inhibitor of metalloproteinases (TIMPs) as a function of age between subjects aged over 30 years old (>30) and subjects 30 years old and younger (≤30). Analysis performed with PRP WBC controlled for as a covariate; *Significant effects *P* < 0.05.

## Discussion

The regulation of wound healing, the cleaving of growth factor receptors, and the modification of the extracellular matrix are several functions of MMPs [9,10]. The purpose of the present study was to assess variations of MMPs and TIMPs from activated, concentrated platelets from normal male blood donors. The major findings of the study included a significant increase in TIMP-2 and TIMP-4 concentrations for individuals aged over 30 compared to individuals 30 years of age and younger. MMP-2, MMP-9, TIMP-1, and ADAMTS13 did not demonstrate any statistically significant changes with age. The shift in the MMP:TIMP balance could potentially lead to a change in the cumulative effect of PRP therapies causing a decreased effectiveness of this modality in healing musculoskeletal pathologies as individuals age.

The use of concentrated, activated platelets or PRP remains popular in the clinical and operative setting despite mixed clinical results. Gardner et al. [1] performed a retrospective analysis including 61 patients undergoing total knee arthroplasty (TKA) with intraoperative use of PRP and compared outcomes of pain, motion, blood loss, and hospital stay to 37 control subjects. The PRP group had less blood loss as measured by preoperative and day 3 postoperative hemoglobin measurements (2.7-g/dL vs. 3.2-g/dL decrease, *P* = 0.026), decreased narcotic use while in hospital, achieved a higher range of motion prior to discharge, and were discharged an average of 1 day earlier compared to the control group.

Other studies for the therapeutic application of PRP demonstrated sustained improvement of symptoms for patients with lateral epicondylitis [13], decreased time to resume training for athletes after open repair of the Achilles tendon [14], and increased functional scores and decreased pain after rotator cuff repair [15]. However, given the paucity of quality randomized controlled trials on this subject, the benefits are called into question upon further review by other authors. A meta-analysis reviewed 15 randomized controlled trials and five prospective cohort studies and found no clear clinical benefit of PRP [5]. There was a trend of benefit favoring PRP use, but this was not statistically significant with wide confidence intervals. Varied clinical results have necessitated further basic science investigations to determine the optimal platelet concentration, preparation technique, and balance of cell signaling molecules to aid healing.

The effect of PRP is likely not due to the action of a single growth factor, cytokine, MMP, or TIMP, but instead a sum of synergistic effects of these dynamic factors during tissue remodeling and healing [16]. MMPs are involved in wound healing and pathological conditions including osteoarthritis and rheumatoid arthritis [17]. MMPs are proteases that degrade gelatin, collagen, elastin, aggrecan, osteonectin, cytokines, growth factors, and receptors [17]. These molecules establish chemotactic gradients, regulate inflammation, and extravasation of leukocytes into injured tissue [9]. MMP-9 (Gelatinase B) is directly implicated in regulating inflammation by modulating cell migration [9].

TIMPs have historically been thought of as inhibitors of MMPs with varying affinities for specific MMPs [9,17,18]. However, it has become clear that TIMP function may be more complex than previously estimated; in some cases, a TIMP may actually activate an MMP. For example, TIMP-2 activates MMP-2 (in complex with active MMP-14) when it binds to the hemopexin-like domain of MMP-2 [19]. In contrast, TIMP-2 inhibits MMP-2 when it binds to the catalytic site of MMP-2 [19]. TIMPs also affect cell proliferation independent of their inhibition effects on MMP activity [18]. Kasper et al. [20] showed that MMPs and TIMPs likely induce mesenchymal stem cells in response to mechanical force at a fracture site. The authors argue that the balance of MMPs and TIMPs is likely the deciding factor, as opposed to the individual activity of any single bioactive molecule. Thus, these molecules must be considered to grasp the overall effects of PRP activity. The selected MMPs, TIMPs, and ADAMTS13 quantified in this study were chosen as a representative sample of these classes of molecules secondary to their release from platelets, biological effects, and reproducible, cost-effective commercial assays available to researchers.

The concomitant expression of MMPs and TIMPs has been documented in gene studies [21]. The reason the protease and its inhibitor are simultaneously induced is for tight control of the extent of extracellular matrix degradation and remodeling [18]. The correlation between MMP and TIMP concentrations in this study is likely for the same reason, but further investigation considering the independent functions of these molecules remains.

In this study, the authors observed a statistically significant increase in TIMP-2 and TIMP-4 with age (*P* = 0.04 and *P* = 0.04, respectively). This shift of balance in the protease:inhibitor ratio with age could alter overall tissue remodeling potential. This issue is made even more complex, given that TIMP-2 has multiple binding sites on MMP-2, at least one of which is required first for protease activation [19]. Only after activation, would the inhibitory effects of TIMP-2 and other TIMPs targets be seen, *via* binding at the catalytic site. This mechanism of enzymatic activation by TIMPs, or pro-protein convertases, is best known with the previous example, but is presumed to occur for other MMPs as well [19]. The authors hypothesize that an increased TIMP-2 and TIMP-4 concentration with age, as was demonstrated in this study, would lead to a change in MMP: TIMP equilibrium, causing an overall decrease of activity of the MMP family. This effect would also impact growth factor activity, chemokine activity, and the inflammation pathway. A decreasing trend in overall MMP activity with age as measured using a generic MMP substrate was observed. The cumulative effect of these components may result in the decreased efficacy of this therapeutic modality as a function of age.

Limitations of this study include the *in vitro* analysis of MMP and TIMP concentrations by ELISA. This is an appropriate technique to quantitate these biomolecules that would be given during therapeutic application of activated, concentrated platelets, but does not measure activity. No therapeutic intervention or patient outcome was evaluated in this study design. Given the male dominant military demographics of the donor population, insufficient female samples were collected to have adequate power for further analysis. Further studies should be conducted to include this population and perform clinical studies in conjunction with PRP composition analysis to correlate with patient outcome.

The selected biomarkers represent a small fraction of all biologically active molecules released from platelets and must be considered during interpretation of this study. The platelet proteome variation is known to change with advancing age [22]. Therefore, other changes in biomarker expression in addition to the findings in this study may have a role in affecting healing. TIMP-1, TIMP-2, and TIMP-4 are known to reside in distinct patches separate from the α-granule that contains many molecules including Platelet Factor 4 (PF4), von Willebrand factor (VWF), clotting factors, and growth factors [23,24]. The kinematics of release from these separate storage areas compared to the α-granule is not yet understood and could be a confounding factor.

## Conclusions

MMPs and TIMPs act on biomolecules to regulate the extracellular matrix during the tissue remodeling process. In this study, TIMP-2 and TIMP-4 increased in concentration with age (*P* = 0.04 and *P* = 0.04, respectively) (Table 3). The overall change of the MMP:TIMP equilibrium could impact the efficacy of PRP therapies. Enhanced understanding of the concentration and balance of these molecules in PRP, coupled with the existing knowledge of growth factor and cytokine contents, will better identify patients whose biology will favor positive clinical results of this therapeutic modality. Further clinical investigation is warranted to confirm these laboratory findings.

## Abbreviations

ADAMTS13: a disintegrin and metalloproteinase with thrombospondin motifs 13; ACP: autologous conditioned plasma; ELISA: enzyme-linked immunosorbant assay; ra: relative activity; IRB: Institutional Review Board; MMP: matrix metalloproteinases; PDGF-AB: platelet-derived growth factor-AB; PDGF-BB: platelet-derived growth factor-BB; PF4: platelet factor 4; PRP: platelet-rich plasm; RBC: red blood cell; rp: Pearson's correlation coefficient; TIMP: tissue inhibitor of metalloproteinase; TKA: total knee arthroplasty; TGF-1: transforming growth factor-β1; VWF: von Willebrand factor; WBC: white blood cell.

## Competing interests

The authors declare that they have no competing interests.

## Authors' contributions

JMH conducted sample collection, laboratory analysis, manuscript writing, final manuscript approval, and submitted the manuscript. JLE conceived of the study, participated in its design and coordination. PCJ conducted sample collection. SDZ conducted laboratory analysis, statistical analysis, manuscript drafting and revisions. MKG collected samples and assisted in manuscript revisions. JCM assisted in study design and manuscript revisions. JAB supervised the study, aided study design, and manuscript revisions. All authors read and approved the final manuscript.

## Disclaimer

The opinions or assertions contained herein are the private views of the author(s) and are not to be construed as official or as reflecting the views of the Department of the Army or the Department of Defense.

We certify that all individuals who qualify as authors have been listed; each has participated in the conception and design of this work, the analysis of data, the writing of the document, and the approval of the submission of this version; that the document represents valid work; that if we used information derived from another source, we obtained all necessary approvals to use it and made appropriate acknowledgements in the document; and that each takes public responsibility for it. Nothing in the presentation implies any Federal/DOD/DON/DOA endorsement. Each author certifies that he or she has no commercial associations (eg, consultancies, stockownership, equity interest, patent/licensing arrangements, etc.) that might pose a conflict of interest in connection with the submitted article. None of the authors received any funding for this manuscript.

This article fits the description in the US Copyright Act of 1976 as a ‘US Government Work’. This article was written as part of our official duties as a government officer(s) or employee(s). Therefore, it cannot be copyrighted. The article is freely available to you for publication without a copyright notice, and there are no restrictions on its use, now or subsequently.
